# Preparation and Properties of the Urea-Formaldehyde Res-In/Reactive Halloysite Nanocomposites Adhesive with Low-Formaldehyde Emission and Good Water Resistance

**DOI:** 10.3390/polym13142224

**Published:** 2021-07-06

**Authors:** Jingbiao Song, Shiwei Chen, Xibin Yi, Xinfu Zhao, Jing Zhang, Xiaochan Liu, Benxue Liu

**Affiliations:** 1 Shandong Provincial Key Laboratory of Processing and Testing Technology of Glass and Functional Ceramics, School of Material Science and Engineering, Qilu University of Technology (Shandong Academy of Sciences), Jinan 250353, China; song.jingbiao@foxmail.com; 2 Shandong Key Laboratory for Special Silicon-containing Material, Advanced Materials Institute, Qilu University of Technology (Shandong Academy of Sciences), Jinan 250014, China; yixb@sdas.org (X.Y.); zhaoxinfu@sdas.org (X.Z.); zhangjing@sdas.org (J.Z.); liuxiaochan@sdas.org (X.L.); liubenxue@sdas.org (B.L.)

**Keywords:** urea formaldehyde resin, halloysite, formaldehyde emission, water resistance

## Abstract

Low-cost urea formaldehyde resin (UF)/reactive halloysite nanotubes (HNTs) nanocomposite adhesive was prepared successfully via in situ polymerization. The HNTs were modified to improve its compatibility with polymer. The XRD and FTIR results showed that physical and chemical interaction between the HNTs and polymer resin influenced the structure of UF owing to the functional groups on the HNTs. It is found from SEM images that the modified HNTs could be dispersed uniformly in the resin and the nanocomposite particles were spherical. The performance experiment confirmed that thermal stability of nanocomposite increased largely, formaldehyde emission of UF wood adhesive reduced 62%, and water resistance of UF wood adhesive improved by 84%. Meanwhile, the content of HNTs on the nanocomposites could be up to 60 wt %. The mechanism of the nanocomposites based on the reactive HNTs was proposed. The approach of the preparation could supply an idea to prepare other polymer/clay nanocomposites.

## 1. Introduction

UF is widely used in plywood, fiberboard, and particleboard. It is a typical thermosetting resin adhesive with large production [1,2,3]. The current global production of UF resins exceeds 5 million metric tons (t) annually. They are widely used in particleboard, medium density fiber board, and interior plywood manufacturing [4,5]. Compared with other wood adhesives such as phenolic resin, melamine formaldehyde resin, and polyurethane, UF has the advantages of low cost, fast curing speed, low curing temperature, easy secondary processing, good panel performance, short press times, colorless glue line, etc. [6,7]. Now, there are three problems to inhibit its application on industry: the first problem is toxic formaldehyde, which could pollute the environment and threaten health. In order to decrease the formaldehyde emission of wood-based panel products, reducing the formaldehyde/urea molar ratio is widely used in industry. However, the content of active hydroxyl groups in UF and the number of hydrogen bonds between UF and wood could significantly reduce, resulting in the decrease of initial viscosity of UF. Lower formaldehyde/urea mole ratios also caused a loss of panel properties, particularly internal bond (IB) strength and reduced modulus of rupture (MOR) [8,9]. Several methods have also been successfully applied in the reduction of formaldehyde emissions in the literature. For example, some scientists added formaldehyde scavengers, bio-particles or using in situ polymerization to reduce the formaldehyde emission [10,11,12,13]. In addition, many researchers try to use melamine or phenol as copolymerization modifier to reduce the formaldehyde emission of UF [14,15,16]. Kim used UF and UF modified by phenol to prepare particleboard. It was found that the formaldehyde emission of UF modified by phenol decreased [17]. A series of UF modified by phenol with good performance can be prepared by adjusting the molar ratio of phenol to urea. This method is important and effective. The products can meet the requirements of low formaldehyde emission in much application area. However, melamine, phenol, and other copolymerization modifiers are petrochemical products, which could cause serious environmental pollution and overload the resource burden as they are widely used [18,19,20]. The seriously environmental problems are against green and sustainable development [21,22,23]. The second problem is that: much flour or starch was used to adjust the initial viscosity and rheological behavior of the resin. This method aimed to increase the molecular weight of wood adhesive based on UF and adjust the initial viscosity and rheological behavior of the resin. Thus discontinuous adhesive layer, which was caused by excessive flow and penetration of the resin after sizing, could be prevented [24]. However, flour or starch for wood-based panel are from food resources, which aggravates the shortage of food resources [25,26,27,28]. Therefore, the development of UF based on non-grain fillers has caused concerned. The third problem is poor water resistance, which restricted largely UF application on humid environment. A variety of modifiers has been applied for the enhancement of UF bonded plywood and particleboard water resistance: polymeric methylene diphenyl diisocyanate, melamine acetate, or small albumin [29,30,31].

In order to solve these problems, it has been reported that mineral powders including nano clay and nano silica are used as UF fillers in scientific research [32,33,34,35,36]. These fillers can prevent the excessive penetration of resin into wood pores, improve the initial viscosity, reduce the internal stress of cured adhesive layer, improve the aging resistance of glued wood-based panels, and reduce the formaldehyde emission [37,38,39]. Among these mineral fillers, HNTs have received great attention in recent years.

HNTs are natural aluminosilicates [40,41,42]. They are widely distributed on the world and are relatively cheap. The outer diameter of HNTs is about 30~50 nm, the inner diameter is about 15–20 nm, and the length varies from 500 nm to 1500 nm. It is generally composed by more than 20 curled layers [43,44]. The specific surface area of HNTs is large. HNTs are easy to cluster together. Since the discovery of HNTs, they have attracted the attention of scholars at home and abroad. Owing to its physical and chemical particularity, it has been more and more widely used in various fields [45,46]. For example, the HNTs with curly tubular structure could be used as an efficient adsorbent especially in the adsorption area [22,47]. Zhao studied the effect of temperature, pH, and initial concentration on the adsorption of methylene blue by HNTs. The results showed that the highest adsorption rate could reach 84.32 mg/g under suitable conditions [48]. Peng L studied the effect of temperature, adsorbent dosage, and other factors on the adsorption of cationic dye neutral red by HNTs. It was found that higher dye concentration and temperature were conducive to the efficient adsorption, and the adsorption isotherm was in line with the Langmuir and Freundlich isothermal adsorption model. The maximum adsorption capacities were 54.85 mg/g (298 K), 59.24 mg/g (308 K), and 65.45 mg/g(318 K), respectively [49].

In order to further expand the lumen volume of HNTs, a small molecule can be activated and intercalated to the lumen, providing opportunities for large molecules to fill in [50,51,52]. The principle is to use amino or hydroxyl functional groups of the intercalating agent to interact with HNTs and replace the original crystal water in HNTs. Thus, the interlayer spacing is increased. The small molecules include formamide, potassium acetate, dimethyl sulfoxide, hydrazine, and urea. For example, researchers intercalated urea into HNTs by ultrasonic treatment. The specific surface area of HNTs increased, which improved the desulfurization rate of HNTs [53,54]. However, the compatibility between pristine HNTs and UF is poor, and these fillers could not be averagely dispersed by simple mechanical stirring. This problem greatly affects the production efficiency and product quality of wood-based panels, which restricted largely its application [55,56,57,58].

Herein, we propose an effective approach to modify HNTs and prepare the urea-formaldehyde resin/reactive halloysite nanocomposite via in situ polymerization. The main aims are as follows: firstly, we expect to prepare the reactive HNTs, which have good dispersion in the polymer matrix; secondly, we try to greatly decrease the formaldehyde emission and improve the water resistance; and finally, we want to discuss the mechanism to prepare the urea-formaldehyde resin/reactive HNTs nanocomposites.

## 2. Materials and Methods

### 2.1. Materials

HNTs of diameter 40–90 nm, while their length 300 nm–2.2 μm were supplied by SanXing High-New Material Company of Zaozhuang, China. 4,4′-Oxidianiline (ODA) and 3,3,4,4-benzophenone tetracarboxylic dianhydride (BTDA) were supplied by Alfa Aesar Company, China. (3-Aminopropyl)triethoxysilane (KH550), formalin (37 wt %), acetone, sodium hydroxide, tetraethoxysilane (TEOS), urea, ammonia (28 wt %) and anhydrous ethanol (99.5%) were of analytical grade and were purchased from Beijing Chemical Reagents Company (Beijing, China). Deionized water was used in all experiments.

### 2.2. Preparation of Reactive HNTs

Firstly, the desired amount of dried HNTs were dispersed in the solution of ethanol (100.0 mL) and ammonia (18.4 mL) and kept stirring for 2 h at room temperature. The suspension was heated to 60 °C, and TEOS (2.0 mL) was added. After stirring for 6 h, the suspension was filtrated and washed with ethanol for several times. Then, the product was dried for 12 h, grinded, and sieved through a 250 um mesh. The product yield was 92% and the product was named T-HNTs.

Secondly, the desired amount of T-HNTs were dispersed in the ethanol (100.0 mL) again and stirred at room temperature. KH550 (2.0 mL) was put in the solution of ethanol (10.2 mL) and water (0.8 mL). After continuous stirring for 1 h, the pH was adjusted to 4–5 using acetic anhydrade. Then, the hydrolyzed KH550 was obtained. Afterward, the hydrolyzed KH550 was added into the slurry above and stirred for another 2 h at room temperature. The slurry was filtrated and washed with ethanol several times. The product was then dried for 12 h, grinded, and sieved through a 250 um mesh. Finally, the reactive HNTs were prepared. The product yield was 93% and the product was termed as TH-HNTs.

### 2.3. Preparation of UF/TH-HNTs Nanocomposites

Urea (3.0 g), formaldehyde (3.6 mL), and deionized water (25.0 mL) were placed in a three-necked round-bottomed flask. The pH of the solution was adjusted to 8–9 with sodium carbonate. The temperature of the solution was heated to 75 °C and kept stirring for 1 h. The solution is named by pre-UF. The desired amount of TH-HNTs, deionized water (100.0 mL), and sodium dodecyl benzenesulfonate (2.0 g) were placed in a three-necked round-bottomed flask and were kept stirring for 2 h at room temperature. Then, the solution was heated to 60 °C and the pre-UF was transferred to the flask. After continuous stirring for 3 h, the slurry was filtered and washed with water for several times. The UF/TH-HNTs nanocomposites were prepared. The product yield was 91%. The product was termed by UF-TH. The product containing TH-HNTs of 20, 30, 40, and 60 wt % was named by UF-TH-20%, UF-TH-30%, UF-TH-40%, and UF-TH-60%, respectively. UF and UF/pristine HNTs nanocomposites containing 20 wt % pristine HNTs were also prepared; they were denoted by UF and UF-H-20%, respectively.

### 2.4. Preparation and Testing of Plywood

Three-layer plywood panels of dimensions 300 mm × 300 mm ×1.5 mm were prepared using eucalyptus veneers. The veneers were dried to 3% moisture content before use. The UF/TH-HNTs nanocomposites were mixed with 5 wt % ammonium chloride solution and 20 wt % starch. Then, the adhesives were applied to both sides of the veneer at a spreading rate of 350 g/m^2^ (to form two gluelines). In order to allow the nanocomposites to penetrate the veneers, the veneers were aged for about 15 min. The veneers were prepressed at 1.0 MPa for 1 min at room temperature, and then hot pressed at 120 °C and 1.5 MPa for 4.5 min (1 min/mm). Then, the panels were cooled and conditioned at 20 °C and (65 ± 2)% relative humidity until the weight was constant. The prepared panels were used to test the formaldehyde emissions and water resistance.

### 2.5. Characterization

Fourier-transform infrared (FTIR) spectroscopy was examined (Spectrum 1000, Perkin-Elmer, Waltham, MA, USA) in the region 4000–400 cm^−1^ with a resolution of 4 cm^−1^ and 32 scans. Specimens were prepared by grinding the sample with KBr. The X-ray diffraction (XRD, Shimadzu-6100, Kyoto, Japan) pattern was performed at 40 kV and 30 mA (1200 W), with filtered Cu K radiation from 5° to 60° and a scanning speed of 20°/min using a Siemens D-500 diffractometer. The images of the samples were examined using scanning electron microscopy (SEM, XL 30, FEI, Hillsboro, OR, USA; accelerating voltage 20 kV). The samples were coated with a thin carbon film. The average molecular weights and the molecular weight distribution of the samples were determined by gel permeation chromatography (GPC) (model PL-GPC 50, Agilent Technologies, Santa Clara, CA, USA). The elution solvent was tetrahydrofuran with a constant flow rate of 1 mL/min. The thermal stabilities of the samples were examined on a TG-differential thermal analysis (DTA) instrument (STA449, Netzsch, Selb, Germany) under nitrogen at a heating rate of 20 °C /min from 25 °C to 700 °C. The sample was placed in an alumina crucible. An empty alumina crucible was used as a reference. Three-layer plywood test specimens prepared in part 2.4 were placed in a 10 L glass desiccator together with a Petri dish filled with 300 mL deionized water. A sample hold was put in the desiccator. Three specimens of dimensions 150 mm × 50 mm ×1.5 mm were fixed on the sample hold. The formaldehyde emission tests were performed for 24 h at 20 °C. The quantity of emitted formaldehyde was determined from the concentration of formaldehyde absorbed by the water. The process was as follows: 10 mL formaldehyde solution, 10 mL acetylacetone (0.4 V%), and 10mL ammonium acetate solution (20 wt %) were put into 50 mL triangular flask with stopper. Then, the flask was put in a constant temperature water bath (40 ± 2) °C. 15 min later, the solution was placed in dark for 60 min at room temperature. The UV visible spectrophotometer (Shanghai Yoke Instrument Co.,Ltd., Shanghai, China) was used to measure the absorbance at 412 nm, and distilled water was used as the reference solution. The absorbance A_s_ of the solution and the absorbance A_b_ of the reference solution were determined. By making standard working curve of formaldehyde solution, the calculation factor of sample, and hence a formula for computing formaldehyde content were obtained.

F = f × (A_s_ − A_b_) (1)

F—formaldehyde emission content, mg/L;

f—the slope of standard working curve, mg/L;

A_s_—the absorbance of the solution;

A_b_—the absorbance of the reference solution.

The results were recorded as average of the values obtained from three specimens for each composition. The relative standard deviation was 2%. Three-layer plywood test specimens prepared in Section 2.4 were placed in boiling water. The cracking time was measured to determine water resistance of UF nanocomposite adhesive. Five specimens of each composition were used for the measurements and average values were reported.

## 3. Results and Discussion

**Figure 1:**
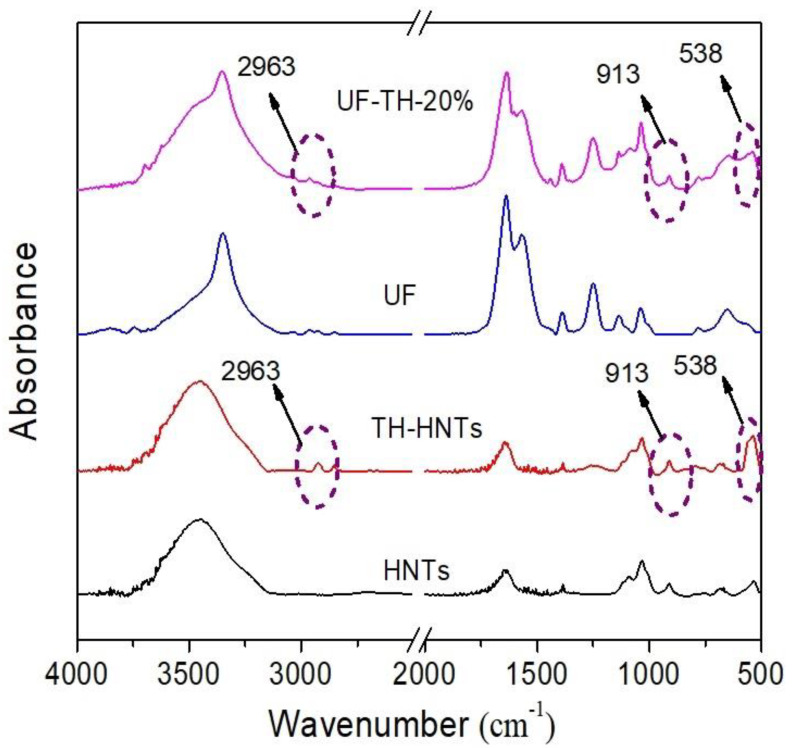
FTIR spectra of HNTs, TH-HNTs, UF, and UF-TH-20%.

Figure 1 showed the FTIR spectra of HNTs, TH-HNTs, UF, and UF-TH-20%. According to Figure 1, the bands at 913 cm^−1^ and 538 cm^−1^ were attributed to Al–O–OH vibration and Al–O–Si vibration of the halloysite. It could be found that there was a new peak at 2963 cm^−1^ in the spectra of TH-HNTs compared to that of HNTs. This peak was attributed to C–H stretching vibration of couple agent. This phenomenon showed that the couple agent was grafted on HNTs [43,59]. According to Figure 1, the peaks at 3350 cm^−1^ and 1637 cm^−1^ were attributed to the N-H stretching vibration and C=O stretching vibration, the peak at 1569 cm^−1^ was ascribed to the N–H bending vibration, and the band at 1248 cm^−1^ was corresponding to the C–O stretching vibration. The peak at 1030 cm^−1^ is the characteristic stretching vibration of C–N groups in the UF. It is noted that there were three new bands at 2963 cm^−1^, 913 cm^−1^, and 538 cm^−1^ in the FTIR spectra of UF-TH-20%. These bands were corresponding to those of TH-HNTs. It could be noted that the band at 3350 cm^−1^ became wide, which could be affected by the chemical or physical interaction between the UF and TH-HNTs.

**Figure 2:**
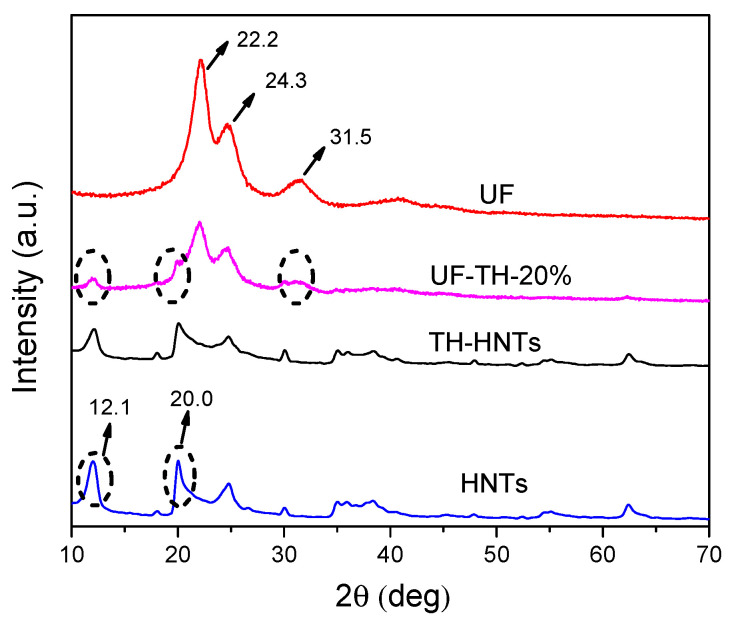
XRD diffraction patterns of HNTs, TH-HNTs, UF, and UF-TH-20%.

The structural characterization of HNTs, TH-HNTs, UF, and UF-TH-20% samples were conducted using XRD. Figure 2 showed the XRD spectra of HNTs, TH-HNTs, UF, and UF-TH-20%. According to the spectra Figure 2, the shark peak at 2θ = 12.1° in the spectra of HNTs and TH-HNTs was attributed to the typical diffraction peak of the HNTs. According to Brag equation, the layer space was 0.73 nm. It could be found that the intensity of peaks at 2θ = 12.1° and 20.0° on TH-HNTs decreased compared to those peaks of HNTs. The reason is probably that the coupling agent may influence the intensity of peaks. According to the spectra Figure 2, the shark peaks at 2θ = 22.2°, 2θ = 24.3°, and the wide peak at 2θ = 31.5° were the typical diffraction peaks of UF. Besides, the typical diffraction peak at 2θ = 12.1°and 20.0° of HNTs occurred in the spectra of UF-TH-20%. It is noted that the peak at 2θ = 31.5° shifted to the low angle. This is probably because the TH-HNTs influenced the UF structure owing to the physical and chemical interaction between the amino or hydroxyl groups of TH-HNTs and those of UF.

**Figure 3:**
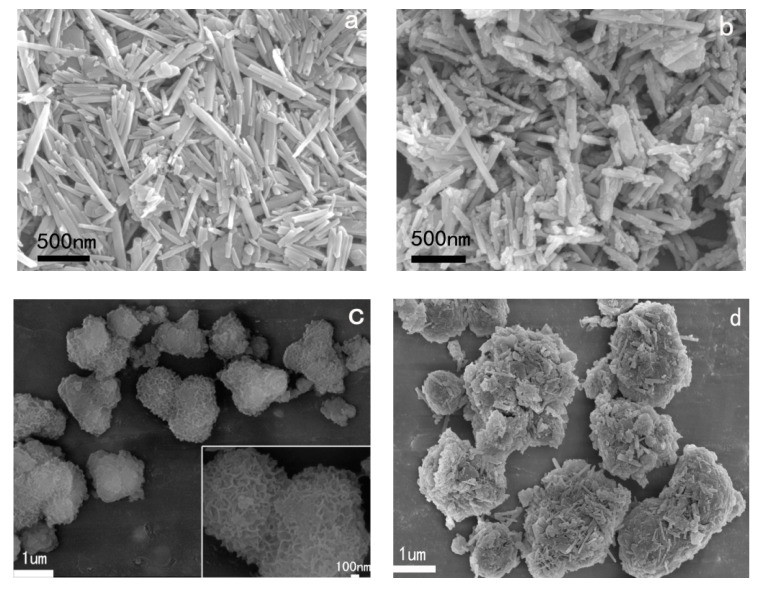

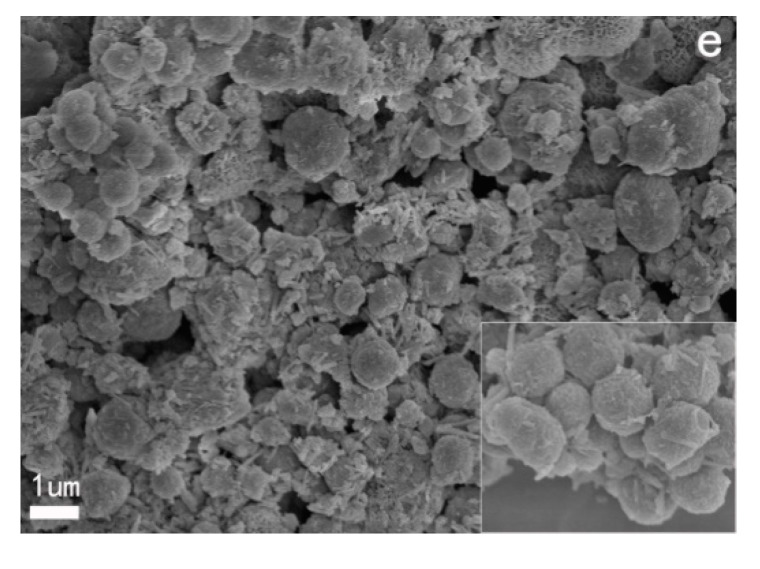
SEM images of (**a**) HNTs; (**b**) TH-HNTs; (**c**) UF; (**d**) UF-H-20%, and (**e**) UF-TH-20%.

Figure 3 showed the SEM images of pristine HNTs, TH-HNTs, UF, UF-H-20%, and UF-TH-20%. The surface of HNTs was smooth, while the surface of TH-HNTs became rough. This is because the coupling agent covered the HNTs [60]. It could be seen from (c) that UF were the irregular particle. The images of UF-H-20% and UF-TH-20% were apparently different from that of UF. It is noted that the UF-TH-20% particles were spherical. Compared to UF-H-20%, TH-HNTs were dispersed more averagely in the polymer resin, showing that TH-HNTs were compatible with polymer more easily.

Table 1 showed the molecular weights and molecular weight distribution of UF and UF/HNTs nanocomposites. It could be seen that the weight average molecular weight Mw and the number average molecular weight Mn of UF-TH-20% were higher than that of UF. This is because that: firstly, the TH-HNTs could easily adsorb formaldehyde and short molecules, so the local concentration increased, which was beneficial to the polymerization and could increase the molecular weight; secondly, the functional group of the TH-HNTs could react with the hydroxyl group of the polymer, which could prolong the polymer chain. It could also be found that the Mw and Mn of UF-TH-20% were higher than those of UF-H-20%. The reason is as follows: firstly, the hydrogen bond between the amino groups of the TH-HNTs and those of UF could easily be formed, which could increase further the adsorption of the small molecules; secondly, the TH-HNTs had better dispersion in the UF, there were fewer large aggregates, so the hindering effect on the molecular movement was weak and monomer or short polymer chain could easily make the reaction, thus prolonging the polymer chain. Besides, Mw/Mn of UF-H-20% and UF-TH-20% were larger than that of UF, showing that the HNTs and the TH-HNTs influenced the molecular weight distribution.

**Figure 4:**
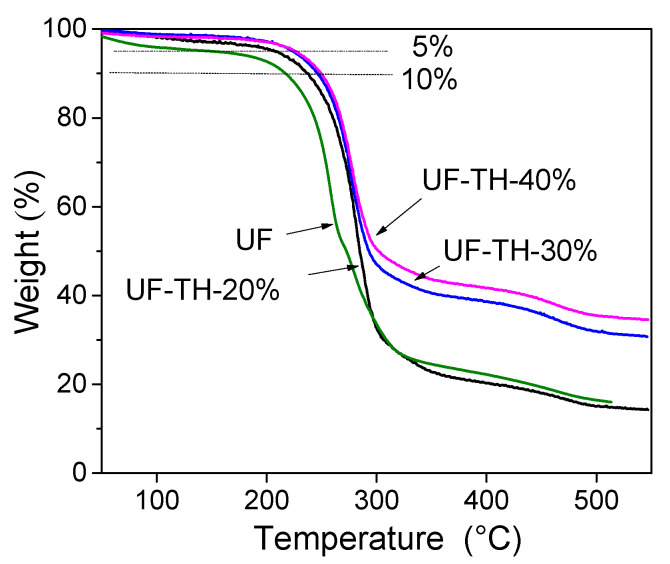
TGA curves of UF and UF/HNTs nanocomposites.

Figure 4 showed the TGA curves of UF and UF/HNTs nanocomposites. According to the TGA curves, the temperature change of UF and UF/HNTs nanocomposites showed the similar trend. There are two main process of weight loss. The first weight loss ranged from 0 °C to 200 °C. Among the temperatures, the weight loss from 50 °C to 100 °C was ascribed to the water evaporation, and the weight change from 100 °C to 200 °C was attributed to formaldehyde evaporation. When the temperature was up to 200 °C, the second weight loss began. The polymer chain was broken, and the network was destroyed.

It could be seen that the temperature at 5% weight loss of UF-TH-20%, UF-TH-30%, and UF-TH-40% was 207.8 °C, 222.3 °C, and 225.2 °C, respectively. The temperature increased by 51.9 °C, 66.4 °C, and 69.3 °C compared to the 5% weight loss temperature 155.9 °C of UF. At the same time, the temperature at 10% weight loss of UF-TH-20%, UF-TH-30%, and UF-TH-40% was 236.8 °C, 247.0 °C and 252.3 °C, respectively. The temperature increased by 18.8 °C, 29.0 °C, and 34.3 °C compared to the 10% weight loss temperature 218.0 °C of UF. The result showed that UF/TH-HNTs nanocomposites had better stability.

It could also be found that the nanocomposites became more stable as the increase of the TH-HNTs. The reason is as follows: firstly, the TH-HNTs have better compatibility with polymer owing to the amino groups on the surface of the TH-HNTs; secondly, the TH-HNTs could absorb heat and prevent heat transfer; and thirdly, the network between the TH-HNTs and UF could hinder the release of small molecule from the polymer, thus prevent the polymer degradation. Besides, more functional groups participated in the polymerization with the addition of TH-HNTs. Thus, the interaction between TH-HNTs and polymer became much stronger.

**Figure 5:**
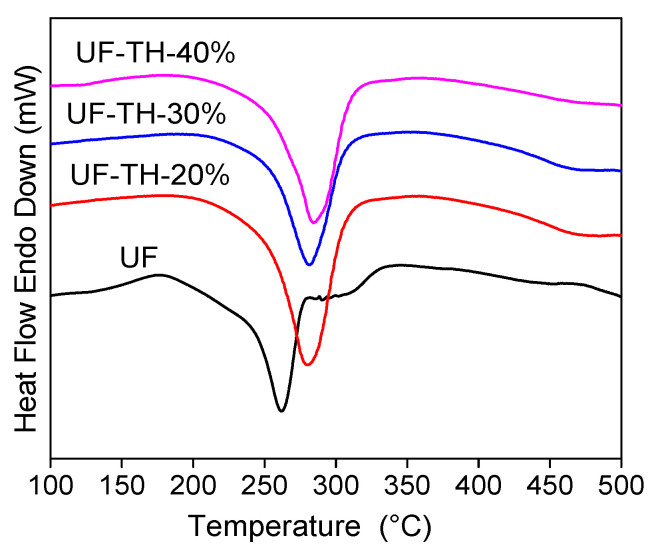
DTA curves of UF and UF/HNTs nanocomposite.

Figure 5 showed the DTA curves of UF and UF/HNTs nanocomposites. When the temperature was above 200 °C, the polymer chain began to be broken. The largest endothermic peak was attributed to degradation of polymer backbone. According to Figure 5, the peak temperature of UF-TH-20%, UF-TH-30%, and UF-TH-40% was 279.2 °C, 281.9 °C, and 284.7 °C, respectively, which was higher than that of UF. The peak temperature of UF was only 261.9 °C. The result showed that the polymer backbone of nanocomposites needed more energy to be destroyed. The reason is as follows: firstly, the TH-HNTs could be dispersed in UF more uniformly and the TH-HNTs were surrounded with UF. The structure of nanocomposites became more compact owing to the hydrogen bond between amino groups of the TH-HNTs and those of UF; secondly, the amino groups of the TH-HNTs could make the chemical reaction with the hydroxyl groups of the UF, thus the cross-linked network formed. The compatible interface between the TH-HNTs and UF occurred. The peak temperatures increased with the addition of the TH-HNTs, showing that the nanocomposites had stronger stability. The result of DTA was consistent to that of TGA.

**Figure 6:**
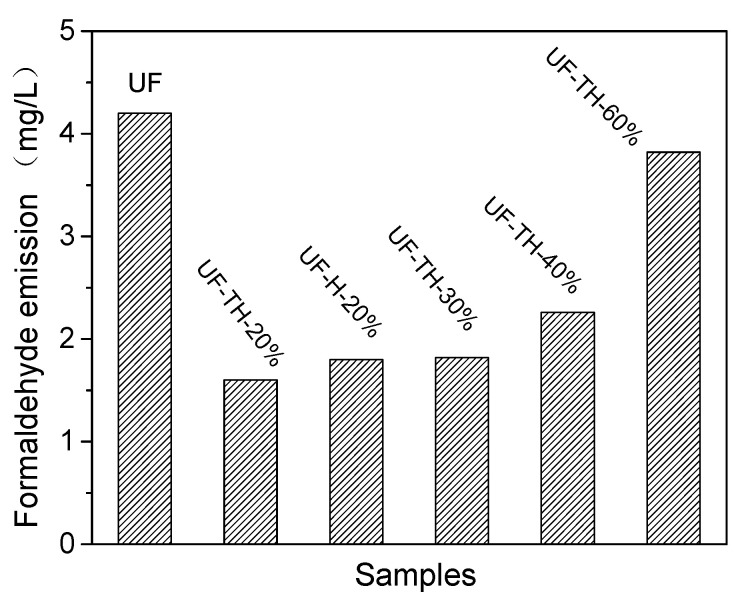
Formaldehyde emission of plywood samples with the addition of UF nanocomposites.

Figure 6 showed formaldehyde emission of plywood samples with the addition of UF nanocomposites. According to Figure 6, UF nanocomposites could effectively decrease the formaldehyde emission of the UF adhesive. After adding the UF-TH-20% to the adhesive, the emission reduced to 1.60 mg/L from 4.20 mg/L. The reason is as follows: firstly, the TH-HNTs could adsorb effectively the formaldehyde; secondly, cross-linking network of the UF nanocomposites could prevent the formaldehyde escaping from the polymer; and thirdly, structural stability of the network became stronger, and the polymer chain could not easily be broken to emit formaldehyde [43]. Compared to the pristine HNTs, the TH-HNTs could more effectively decrease the formaldehyde emission. Meanwhile, it could be noted that the formaldehyde emission was the least when the UF-TH-20% was used. The possible reason was as follows: when the content was 20%, the particles were dispersed averagely in the solution and it was easy to be modified. When the content was too high, above 20%, it was more difficult for the particles to be dispersed in the solution. Thus, it was hard to be modified by the coupling agent. The modified particles had worse compatibility with the polymer. There were more large aggregates, and the hindering effect on the molecular movement was strong. The monomer and short polymer chain could difficultly meet each other to make the reaction. It was hard to prolong the polymer chain. The crosslinking degree of the polymer resin decreased, and the structural stability of the network became weaker, so it was easier for the polymer chain to be degraded and the formaldehyde to be released.

These results are in agreement with other research, where HNTs could be used as adsorbent for small molecules [47,49].

The reduction of formaldehyde emission of the adhesive with the UF-TH-20% was 61.9%. This was higher than 27.9% from another report [11].

**Figure 7:**
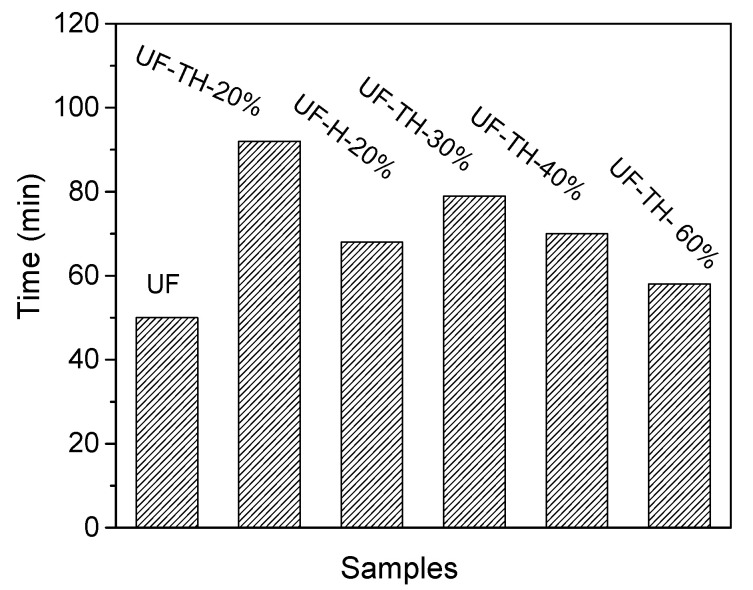
Water resistance of plywood samples with the addition of UF nanocomposites.

Figure 7 showed water resistance of plywood samples with the addition of UF nanocomposites. It could be seen that the water resistance time increased to 92 min from 50 min after adding the UF-TH-20%. The result showed that the nanocomposite improved the water resistance of UF adhesive. The reason that the water resistance was improved is due to the increased hydrophobicity of the adhesive and the reduced water penetration into the bond line. Firstly, the amino group on the TH-HNTs could make the chemical reaction with hydroxyl groups of the polymer resin, thus decreasing the number of hydroxyl groups; secondly, after introducing TH-HNTs, cross-linking degree of the polymer resin increased and the structural stability of the network became stronger, the structure of the particleboards could prevent the penetration of water, so it is more difficult for the polymer chain to be degraded affected by the water [61,62]. Meanwhile, the nanocomposite with the TH-HNTs made the UF better water resistance compared to pristine HNTs.

The water resistance was reduced by 45.7%. This percentage was higher than 35% reported by Hosseyni [63].

It is noted that the water resistance of the samples decreased as the increase of the TH-HNTs. The reason is that more HNTs could agglomerate to large particles, and it was more difficult for the particles to be dispersed in the solution and to be effectively modified by the coupling agent. The modified particles had worse compatibility with the polymer. Owing to the hindering effect of large aggregates on the molecular movement, the increase of the polymer chain was influenced. The crosslinking degree of the polymer resin decreased, and the structural stability of the network became weaker, so the water resistance of the nanocomposites decreased. In addition, more defect interface occurred between the aggregates and the polymers or within the aggregates. The defect interface could store more water, thus the water resistance of the nanocomposites further decreased [25,50,60].

**Figure 8:**
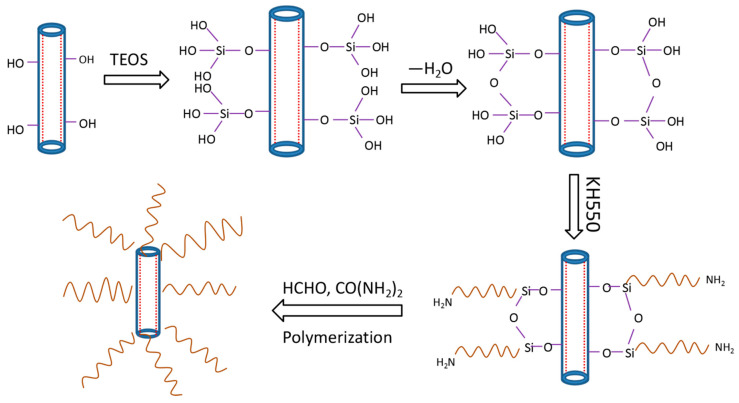
The mechanism to the formation of UF/TH-HNTs nanocomposites.

The mechanism to the formation of UF/TH-HNTs nanocomposites (Figure 8) was proposed based on the results of FTIR, XRD, SEM, etc. When TEOS was used to modify the HNTs, hydrolyzed TEOS could introduce more Si-OH groups to the surface of the HNTs, which was beneficial to the coupling reaction and thus more KH550 was grafted to the HNTs. Owing to the hydrogen bond, the amino group on the TH-HNTs could adsorb formaldehyde to the surface, which could make the polymerization. Thus, the network occurred and the TH-HNTs were covered by the polymer. Finally, the UF/TH-HNTs nanocomposite was prepared. The physical and chemical interaction between the amino groups of the TH-HNTs and the amino or hydroxyl groups of the polymer resin made the network much stronger. Therefore, the compact network could effectively prevent the formaldehyde emission, hinder the degradation of the resin, and thus improve the thermal stability.

## 4. Conclusions

UF/TH-HNTs nanocomposites were successfully prepared, and the HNTs content could be up to 60 wt %. The nanocomposites were spherical particles. The weight average molecular weight Mw and the number average molecular weight Mn of UF-TH-20% were higher than that of UF. Compared to UF, UF/TH-HNTs nanocomposite exhibited better stability. The stability became much stronger with the increase of TH-HNTs. The UF/TH-HNTs nanocomposite could effectively decrease formaldehyde emission and improve the water resistance. After adding UF-TH-20%, the formaldehyde emission of UF decreased to 1.60 mg/L from 4.20 mg/L. The water resistance time of UF increased to 92 min from 50 min. The limitation of this research is that it need much time to modify the halloysite, thus the work is inefficient. The next research is to study efficient method to modify the halloysite.

## Figures and Tables

**Table 1:** The molecular weights and molecular weight distribution of UF and UF/HNTs nanocomposites.

Samples	Mw	Mn	Mw/Mn
UF	23,115	22,359	1.03
UF-H-20%	25,633	22,510	1.14
UF-TH-20%	28,294	22,704	1.25

## Data Availability

The data presented in this study are available on request from the corresponding author.
